# Translation and cross-cultural adaptation of WHOQOL-HIV Bref among people living with HIV/AIDS in Pakistan

**DOI:** 10.1186/s12955-021-01693-0

**Published:** 2021-02-08

**Authors:** Ali Ahmed, Muhammad Saqlain, Nasim Akhtar, Furqan Hashmi, Ali Blebil, Juman Dujaili, Malik Muhammad Umair, Allah Bukhsh

**Affiliations:** 1 School of Pharmacy, Monash University, Jalan Lagoon Selatan, Bandar Sunway , 47500 Subang Jaya, Selangor Malaysia; 2 Department of Pharmacy, Quaid I Azam University Islamabad, Islamabad, Pakistan; 3 Infectious Diseases Department, Pakistan Institute of Medical Sciences, Islamabad, Pakistan; 4 University College of Pharmacy, University of the Punjab, Allama Iqbal Campus, Lahore, 54000 Pakistan; 5 National AIDS Control Programme of Pakistan, Islamabad, Pakistan; 6 Institute of Pharmaceutical Sciences, University of Veterinary and Animal Sciences, Lahore, Pakistan

**Keywords:** Cross-cultural, HRQol, Pakistan, Reliability, Translation, Validity

## Abstract

**Background:**

Reliable Health-Related Quality of Life (HRQoL) assessment will be useful in identifying health issues and in identifying health care actions. Due to the lack of a psychometrically valid tool in Urdu, we aim to translate and examine the psychometric and cross-cultural adaptation of WHOQOL HIV Bref among people living with HIV/AIDS (PLWHA) in Pakistan.

**Methods:**

The standard forward-backwards translation technique was used to convert English version of the WHOQOL HIV Bref into Urdu. After cognitive debriefing, final Urdu version of instrument was developed. Based on the principle of at least 5 subjects for each item, a sample of 182 patients was used using a universal random sampling technique from the Pakistan Institute of Medical Sciences, Islamabad. The Cronbach’s alpha and intra-class correlation coefficients (ICC) were estimated to assess internal validity and reliability of the translated version. Exploratory factor analysis was carried out to determine the factor structure and independent associations between the instrument domains and CD-4T-cell count were assessed using multivariable linear regression

**Results:**

High Cronbach alpha 0.93 was found for all WHOQOL HIV Bref facets. The test–retest reliability demonstrated a statistically significant ICC ranged from 0.88 to 0.98 (*p* < 0.001). In known group validity, lower CD-4 lymphocytes count was significantly related to poor scores for all six domains (*p* < 0.001). Similarly, symptomatic subjects had significantly lower scores compared to asymptomatic subjects on the physical, psychological, social relationship and independence domains (*p* < 0.05). Statistically significant positive correlation of all six domains of instrument with CD4 cells count (*p* < 0.001), exhibiting patients with higher CD-4 cells will have higher mean scores of all domains. Factor analysis revealed 5 domains, including physical health, psychological health, social relationship, environmental, and spiritual health. Multivariable linear regression analysis reported; only physical, psychological health and environment health domains were found significantly associated with higher CD-4 lymphocytes count (Beta = 0.121, *p* < 0.001, Beta = 0.103, *p* = 0.002, and Beta = 0.032, *p* = 0.032).

**Conclusion:**

Findings suggested that the Urdu version of WHOQOL HIV Bref is a psychometrically valid and culturally well-adapted HRQoL measurement tool for PLWHA in Pakistan.

## Background

As per the Joint, United Nations Programme on Human Immunodeficiency Virus (HIV) infection and Acquired Immunodeficiency Syndrome (AIDS) (UNAIDS) estimates, more than 38 million people are living with HIV/AIDS (PLWHA) worldwide [1]. Despite the fall in peak of new HIV cases all over the world the low-middle-income countries (LMICs) like Pakistan are facing an escalation in the number of HIV cases [2, 3]. With the advent of safe Highly Active Antiretroviral Therapy (HAART), the survival of PLWHA has been improved like normal persons [4]. However, Health-Related Quality of Life (HRQoL) of PLWHA remains substantially lower than the general population, even when the majority of HIV-positive people have viral control and are immunologically stable [4–6]. Nevertheless, HIV infection and its associated factors like aging, treatment adherence, social conditions, relationship problems, comorbidities, and social stigma still have a significant impact on (HRQoL), even among individuals whose viral count suppressed by ART [4, 7]. Therefore, HRQoL of PLWHA has become an important focus for researchers and healthcare providers.

HRQoL and other patient-reported outcomes are extremely important for assessing PLWHA experience and perspective [8]. They reflect the satisfaction of patients and the benefit of HAART that is not necessarily covered by other endpoints. HRQoL results are commonly used in clinical trials and regulatory and reimbursement agencies have begun to request these data in their intervention assessment process [9]. Precise evaluation of HRQoL with valid measures has become crucial for improving PLWHA quality of life [10, 11]. Several tools, both generic and disease-specific, are used to measure HRQoL in PLWHA [4]. Generic questionnaires such as the frequently used EQ-5D, WHOQOL, HUI, M-QOL, SF-12, and SF-36 have the advantage of enabling a comparison of HRQoL across different disease populations. However, HIV-specific instruments such as WHOQOL-HIV Bref, HIV-QL-31, MOS-HIV, ACTG-21, AIDS-HAQ, MQOL-HIV, FAHI, PROQOL-HIV, and HIV-SQUAD, have shown greater sensitivity to detect small but clinically significant differences in treatment effects because they focus more on the specific effects of the disease [4, 12].

Originally, generic WHOQOL-100 item scale was developed by World Health Organization (WHO) to assess individuals' perceptions of their position in life in the context of the culture and value systems in which they live and in relation to their goals, expectations, standards and concerns [13]. Then, an abbreviated 26-item WHOQOL Bref has been developed. Later, a 100-item HIV-specific questionnaire (WHOQOL-HIV) was developed and most recently a brief version was developed. The WHOQOL HIV Bref is considered as the most sensitive and reliable particular tool for assessing the HRQoL of PLWHA [10, 14]. It contains both generic measures and HIV specific facets. In Pakistan, a validated version of the generic WHOQOL instrument is available [15]. However WHOQOL HIV bref is not validated in local settings of Pakistan. To be used in a specific country, Such an instrument must be translated and validated in the local language to position the individual HRQoL within their local context in terms of culture and values [16]. This instrument has not been translated into Urdu, although it has been translated into many other languages [12, 16–26]. Hence, the current study aimed to assess the psychometric properties and cultural adaptability of the Urdu version of WHOQOL-HIV Bref in PLWHA in Pakistan.

## Methods

### Ethical considerations

The study protocol was approved by the National AIDS Control Program of Pakistan (NACP) and Research Ethics Committee (REC) of ART center of Pakistan Institute of Medical Sciences (PIMS) (Approval No; 1827). Written informed consent from patients was taken after providing them with the written information as well as a verbal explanation of the study. Participants were told that study participation was voluntary and can be terminated at any time. All the information collected in this research will be confidential and only available to the research team that manages the data; Upon completion of the analysis, the contact details of the participants will be removed. All the study procedures were handled following the Helsinki Declaration ethical principles [27].

### Study design and study setting

For data collection, we adopted a cross-sectional study design. The study was carried out at the ART center at PIMS hospital, which is a 947-bed hospital located in Islamabad Capital Territory (ICT), Pakistan. PIMS hospital is a teaching and referral center catering to the needs of more than 1.5 million population. ART center PIMS is the biggest center of HIV care in Pakistan having more than 4000 registered PLWH who are getting free of charge HIV treatment. Almost 15 to 20 PLWHA visit the ART center daily for their medical needs. This center was selected because of the geographic location, and it is the biggest referral center where patients from different ethnic groups and regions of Pakistan receive their ART.

### Sample size

For the psychometric evaluation of questionnaires, a minimum of five subjects should be selected for each question [28]. Therefore, 155 PLWHA was recruited in the current study for the validation of the 31-item instrument in the present study. With a drop-out rate of 20% (such a high rate was considered due to stigma among PLWH), 190 subjects were approached. Participants who did not complete the questionnaire and patients with missing information (viral load, CD4 T cell count) were excluded; therefore, 182 (95.8%) subjects were included in the final analysis.

### Participants

PLWHA included if they were (1) older than 18 years of age; (2) more than six months of HIV diagnosis; (3) CD-4T lymphocyte count and viral load tests not more than four weeks older; (4) regular follow-up at the ART centre and (5) able to communicate in the Urdu language. Patients who were terminally ill, visually impaired, hearing impaired, multiple comorbid, cognitively impaired, and unable to complete interviews were excluded.

### Questionnaire

The questionnaire consisted of three parts, including sociodemographic information, HIV related characteristics, and the WHOQOL HIV Bref. The sociodemographic part consisted of gender, age, education, marital status, and work status. HIV related components included HIV since diagnosed, viral load, CD-4 lymphocytes count, HIV serostatus, total time on ART and mode of HIV transmission. HIV serostatus was divided into three stages Asymptomatic, symptomatic and AIDS converted similarly CD-4T lymphocytes count were also divided into three groups.

The WHOQOL HIV Bref consisted of a total of 31 questions, including two general questions and 29 specific questions explaining six areas of quality of life. The first domain; physical wellbeing, consisting of four questions, i.e. 3, 4, 14, 21; the second domain; psychological health, consisting of five questions 6, 11, 15, 24, 31; the third domain is the degree of freedom, consisting of four questions 5, 20, 22, 23; the fourth domain is social relations, consisting of four questions 17, 25, 26, 27; the fifth domain is environmental health, consisting of eight questions 12, 13, 16, 18, 19, 28, 29, 30; Sixth domain is spiritual wellness comprised of four questions 7, 8, 9, 10 [8].

Five-point Likert scale is used to develop the ratings ranging from 1, Very poor/Not at all/Very dissatisfied/Never to 5, Very good/Extremely/Completely/Very satisfied/Always. Out of the 31 items, 7 are negative statements, for which responses were reversely coded to ensure higher scores representing better quality of life. Each domain item contributes equally to the score of the domain. Each domain consisted of a different number of questions and the average domain score was multiplied by four for the calculation of each domain score. The total domain score ranged from 4 as the lowest score to 20 as the most effective score [29]. WHOQOL HIV user manual also provides sociodemographic and current health characteristics of PLWHA such as age, gender, marital status and route of HIV transmission [30].

### WHOQOL HIV Bref instrument translation

**Fig. 1:**
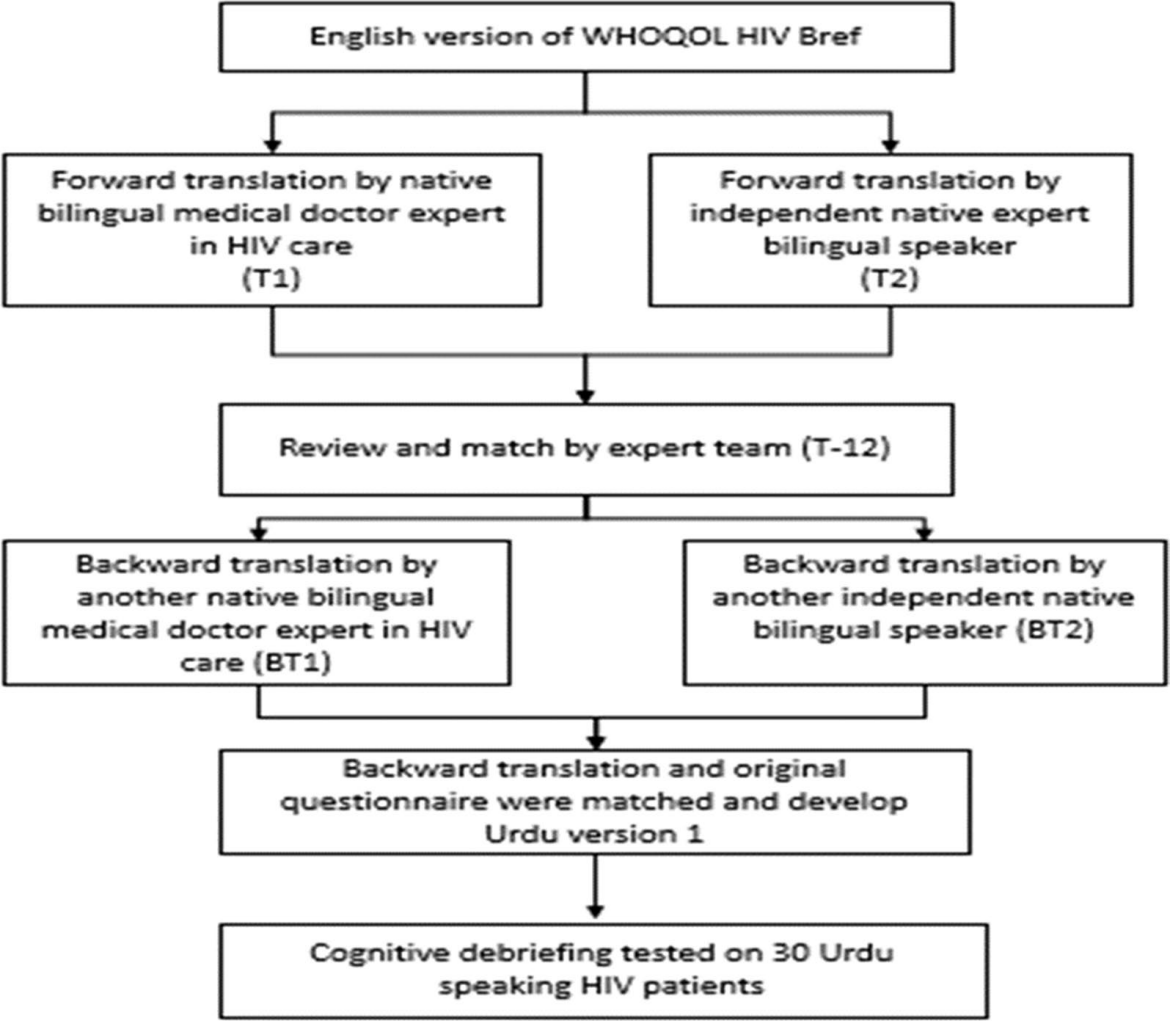
Translation procedure

WHO was contacted regarding permission to conduct cross-cultural validation of WHOQOL HIV Bref in the Urdu language (the national language of Pakistan); the English version of the WHOQOL HIV Bref instrument was translated to Urdu by standardized forward-backwards translation procedure (Fig. 1) explained in 5 steps by Beaton’s guidelines [31]. Expert committee (Comprised of health professionals, language professionals, methodologists and translators) was responsible for the consolidation of original questionnaire and all versions of translation (T1, T2, T12, BT1, BT2). The original English version was independently forward translated into Urdu by a bilingual physician (T1) and a non-medical linguist (T2). Later, the authors reconciled the two translations in order to produce a single version of the forward translation (T12). Later the Urdu version (T12) was sent to native expert bilingual physician (BT1) and a native independent bilingual liguist (BT2) for backward translation. Translators were asked to report any sort of difficulty during translation. After that authors compared the forward backwards translations and amended the questionnaire to produce preliminary translated Urdu version. Upon ensuring consistency in the translated version and the original English version, the preliminary version of Urdu was pre-tested for cognitive debriefing on 30 conveniently chosen PLWHAs at the PIMS hospital. Data were analyzed and Cronbach's alpha for each domain was shown to be between 0.78 and 0.92. Relevant changes to a few items have been made Based on the feedback of the respondents to submit the final version of WHOQOL HIV Bref in Urdu. Expert committee ensured the semantic, idiomatic, experiential and conceptual equivalence of translated version to the original questionnaire.

### Data collection procedure

In PIMS ART center, PLWHA regularly visits for ART follow up (All medicines provided to patients were free of cost from the Government of Pakistan) in a separate counseling room. A simple random sampling technique was used to collect data from PLWHA. Data were collected from July to Sep 2019. Eligible patients participated in the study after written consent under the guidance of a trained investigator. After describing the objectives of the study and nature of research study participants were interviewed face-to-face. Respondents were asked to provide sociodemographic information and rate 31 statements of quality of life instrument describing specific behaviors related to HIV care during the past two weeks. Their clinical data was retrived from their medical recods. They were asked to fill the questionnaire for the collection of sociodemographic information and HIV characteristics. The completed questionnaires were checked by the study investigator and soring were performed. Furthermore, incomplete questionnaires were excluded from the study. Fifty four subjects were asked to complete the WHOQOL HIV Bref questionnaire again after the gap of two weeks for test retest reliability analysis.

### Statistical analysis

Statistical analysis was performed using Statistical Package for Social Sciences version 24® (SPSS v24, SPSS Inc., Chicago, IL, USA). Categorical parameters were calculated as frequencies and percentages, while numerical variables were reported as means and standard deviations. The reliability of the WHOQOL HIV Bref was measured by Cronbach’s α coefficient. Cronbach’s α > 0.70 indicates a good consistency [32]. For test–retest analysis, an Intraclass Correlation Coefficient (ICC) was used with an ICC > 0.75; demonstrating high test–retest reliability [33].

Known group analysis was used to assess how well the Urdu version of the WHOQOL HIV Bref instrument differentiates between patients living with HIV concerning their HIV stages and CD-4T cell count. One-way Analysis of Variance (ANOVA) was performed to assess known-group validity. Post hoc tests were conducted to look at critical contrasts in domain scores among the three CD-4T-cell count /mm^3^ (good CD-4T cell count (≥ 500), medium CD-4T cell count (200–499) and poor CD-4T cell count (≤ 200). It was hypothesized that HIV-symptomatic members and PLWHA with lower CD4 T Cell counts would have lower HRQoL scores mostly. Convergent validity was determined by measuring average variance extract (AVE), and composite reliability (CR) by using smart PLS inbuild command for all facets with minimal acceptable value of 0.5 and value > 0.7 indicates excellent validity [34, 35]. Statistical examinations of all HRQoL items were conducted by utilizing inverted item scores, and a *p* value of < 0.05 (two-tailed test) was considered as a basis of measurable centrality for all analyses [36]. The independent associations between the domains and CD-4T-cell count were assessed using multivariable linear regression [37] . The construct validity was evaluated by Exploratory Factor Analysis (EFA) by considering Kaiser-Meyer Olkin and Bartlett’s test of sphericity. The EFA validity was tried by extracting components via principal component analysis, taken after Varimax rotation with Kaisers’ normalization [38].

## Results

### Patient characteristics

Sociodemographic and health-related data of participants is given in Table 1. Of the total 182 patients, most respondents were males (n = 134, 73.6%), more than half (n = 104, 57.1%) fall in the age category of 25–50 years, and almost half of individuals (n = 90, 49.5%) were married or in a relationship. The majority of participants were illiterate (56%) but were able to understand and converse in the Urdu language. No PLWH had AIDS and 81% were asymptomatic. The majority of participants (53%) infected with HIV through intravenous drug use, while (16.5%) having sex with a male partner is the reason why they got the infection.

**Table 1:** Sociodemographic and clinical characteristics of the participants (n = 182)

Parameters	n (%)
*Gender*	
Male	134 (73.6)
Female	42 (23.1)
Transgender	6 (3.3)
*Age (years)*	
< 25 years	30 (16.5)
25–50 years	104 (57.1)
> 50 years	48 (26.4)
*Marital status*	
Single	54 (29.7)
Married/in relationship	90 (49.5)
Divorced/separated	14 (7.7)
Widowed	24 (13.2)
*Level of education*	
Illiterate	102 (56)
Primary	50 (27.5)
Secondary	12 (6.6)
Tertiary	18 (9.9)
*Employment status*	
Employed	102 (56)
Unemployed	80 (44)
*Since HIV diagnosed*	
< 1 year	44 (24.2)
1–5 years	80 (44.0)
6–10 years	28 (15.4)
> 10 years	30 (16.5)
*HIV serostatus*	
Asymptomatic	148 (81.3)
Symptomatic	34 (18.7)
AIDS Converted	0 (0)
*CD-4T cells count*	
< 200	32 (17.6)
200–500	62 (34.1)
> 500	88 (48.4)
*Viral load*	
Detectable	64 (35.2)
Not detectable	118 (64.8)
*Time on ART (months)*	
< 12	42 (23.1)
12–48	14 (7.7)
> 48	126 (69.2)
*How you got infected with HIV*	
No idea	20 (11.0)
Blood products	20 (11.0)
Injecting drugs	96 (52.7)
Sex with women	16 (8.8)
Sex with man	30 (16.5)

### WHOQOL HIV Bref scale

The WHOQOL HIV-Bref domain scores were calculated following the user’s manual of the WHOQOL-HIV instrument [30]. Questions showing the lowest scores were activities of daily living, sleep and rest, participation in leisure activities, meaningful life and social inclusion. Across the domains, physical health and psychological health showed the highest score; social relationships showed average scores while the level of independence showed the least count. The majority of items have skewness and kurtosis coefficients within the range of − 1.00 to 1.00. Only five items have a coefficient higher than 1.00, but still within the acceptable range of − 2.00 to 2.00 [39]. An item with the highest kurtosis (5.3) was the one measuring friends' social support accompanied by negative feelings (4.5) (Table 2).

**Table 2:** Mean, standard deviation, skewness, and kurtosis of WHOQOL-HIV Bref items

	Mean	SD	Skewness	Kurtosis	Cronbach's Alpha if Item Deleted
*Overall QoL*					
How would you rate your QoL?	3.61	.85	− .584	− .321	0.931
How satisfied are you with your health?	3.67	.84	− .550	− .218	0.931
*Physical health*	15.63				
Pain and discomfort	4.14	1.02	− .913	− .409	0.930
Bothered by HIV symptoms	4.04	.95	− .946	.441	0.930
Energy and fatigue	3.49	.90	− 1.055	.803	0.931
Sleep and rest	3.95	.81	− .543	− .052	0.932
*Psychological health*	15.33				
Positive feelings	2.95	.74	− .094	.146	0.932
Concentration ability	2.49	.90	.016	− .778	0.933
Bodily image and appearance	3.41	.80	− .999	.390	0.931
Self-satisfaction	2.77	.85	.219	− 1.085	0.931
Negative feelings	1.21	.48	2.293	4.588	0.934
*Level of independence*	8.04				
Dependence on medical treatment	4.09	.93	− .749	− .378	0.930
Mobility	4.33	.98	− 1.400	1.074	0.930
Activities of daily living	3.56	.61	− .804	.031	0.931
Work capacity	3.35	.76	− .693	− .149	0.931
*Social relationships*	12.34				
Social acceptance (Inclusion)	1.99	.83	.598	− .119	0.935
Personal relationships	2.11	.58	− .017	− .123	0.931
Sexual satisfaction	1.98	.53	− .021	.546	0.932
Social support from friends	1.97	.62	1.141	5.398	0.934
*Environmental health*	9.02				
Physical safety and security	3.45	.73	− .766	− .482	0.931
Physical environment	3.52	.70	− .734	− .145	0.931
Financial resources	2.60	.87	− .026	− .709	0.931
Information for daily living	3.21	.73	− .351	.781	0.934
Participation in leisure activities	3.00	.82	.118	− .081	0.934
Home environment	2.51	.65	− .780	− .124	0.931
Accessibility of health services	3.19	.72	− .651	.288	0.932
Transport	3.21	.80	− .526	.388	0.931
*Spirituality*	10.27				
Meaningful life	2.88	.74	− .297	− .116	0.933
Forgiveness and blame	2.15	.92	.781	.262	0.933
Concerns about the future	2.02	.59	.314	.924	0.934
Worry about death	1.97	.79	1.000	1.876	0.935

### Reliability test

Reliability analysis revealed excellent reliability of the translated Urdu version of the scale with Cronbach’s alpha value 0.934. All the six subscales exhibited remarkable internal consistencies ranging from Cronbach’s alpha value of 0.931 for the physical health domain to 0.934 for the social relationship domain (Table 3).

**Table 3:** Reliability test; internal consistency and test–retest reliability

Parameters	Cronbach’s alpha (n = 182)	ICC (95% CI) (n = 54)
*Overall*	.934	
Physical	.931	0.975 (0.957–0.986)
Psychological	.934	0.992 (0.986–0.995)
Independence	.932	0.892 (0.870–0.941)
Social Relationships	.934	0.901 (0.880–0.932)
Environment	.932	0.872 (0.850–0.890)

### Test re-test analysis

The test–retest reliability analysis was performed on 54 PLWH after two weeks gap. ICC value > 0.75 (*p* < 0.001) was considered significant [40]. Findings revealed statistically significant (*p* < 0.001) ICC value for all six domains with highest ICC value of 0.99 (95% CI: 0.986–0.995) for psychological domain, and lowest ICC value of 0.872 (95% CI: 0.850–0.890) for environment domain (Table 3).

### Known group validity

Results revealed remarkable differences in the domain’s mean scores stratified by CD4 T cell level. The results indicate patients with higher CD4 T cell count had higher mean scores of physical health, level of independence, psychological health, social relationship, environment health and spiritual health (*p* < 0.001) compared to patients with CD4 T cell level 200–499 cells/mm^3^ and less than 200 cells/mm^3^. Post hoc analysis also demonstrated a statistically significant variation in mean scores for all six domains except spirituality domain; mean count did not vary substantially among patients with CD4 T-cell count less than 200 and patients with CD4 T-cell count 200–499 cells/mm^3^ (Table 4).

**Table 4:** Known-group validity for subcategories of patients by CD-4T cell count/mm^3^ (n = 182)

Categories	CD4 count 200	Sign ^a^	CD4 count 200–499	Sign ^b^	CD4 count 500	Sign ^c^	ANOVA *p*
	n = 32		n = 62		n = 88		n = 182
Physical	10.62 ± 3.01	< 0.001	15.83 ± 2.11	< 0.001	17.31 ± 1.40	< 0.001	< 0.001
Psychological	7.7 ± 2.11	< 0.001	10.09 ± 1.24	< 0.001	11.32 ± 1.36	< 0.001	< 0.001
Independence	11.43 ± 2.27	< 0.001	15.48 ± 1.82	0.002	16.63 ± 1.84	< 0.001	< 0.001
Social relationships	7.18 ± 1.57	0.031	8.06 ± 1.19	0.545	8.34 ± 1.67	0.001	< 0.001
Environment	10.53 ± 2.11	< 0.001	12.50 ± 1.74	0.353	12.89 ± 1.38	< 0.001	< 0.001
Spirituality	8.06 ± 2.57	0.469	8.61 ± 2.05	0.010	9.65 ± 1.81	0.001	< 0.001

Data are M ± SD. Tests were One-way ANOVA and Scheffé Test for post-hoc group comparisons

ANOVA, Analysis of Variance

^a^Regards comparison between the first and second group

^b^Regards the comparison between the second and third group

^c^Regards the comparison between the third and first group

Five out of the six domain scores discriminated significantly among disease stages (asymptomatic and symptomatic). Asymptomatic patients had higher scores acros all subdomains except for spirtuality compared to symptomatic patients (Table 5).

**Table 5:** Comparison of Urdu version of WHOQOL-HIV Bref domain scores between participants with asymptomatic and symptomatic HIV status

Domain	Asymptomatic n = 148 Mean and SD	Symptomatic n = 34 Mean and SD	Mean difference (95% CI)	*p* value
Physical	16.71 (1.95)	10.94 (2.98)	5.77 (4.95–6.59)	< 0.001
Psychological	16.19 (1.85)	11.58 (2.51)	4.60 (3.68–5.52)	< 0.001
Independence	8.26 (1.50)	7.11 (1.47)	1.14 (0.57–1.70)	< 0.001
Social relationships	12.83 (1.58)	10.24 (1.48)	2.60 (2.01–3.18)	< 0.001
Environment	9.26 (2.07)	8.00 (2.17)	1.26 (0.47–2.04)	< 0.001
Spirituality	10.8541 (1.49931)	7.7176 (1.76)	3.13 (2.55–3.72)	0.002

All significant with *p* < 0.05

### Convergent validity

Results revealed that based on the Composite reliability (CR) value, all facets show good convergent validity as CR value is higher than 0.7 except for the social relationship domain 0.664 (0.458–0.755). Other parameters Average Variance Extracted (AVE) also demonstrates that all the facets exhibit acceptable convergent validity (Table 6).

**Table 6:** Convergent validity of six domains of WHOQOL-HIV Bref scale

Domains	Composite reliability (CR)	Average variance extracted (AVE)
Environmental health	0.825 (0.782–0.855)***	0.586 (0.332–0.636)***
Level of independence	0.881 (0.852–0.905)***	0.649 (0.592–0.705)***
Physical health	0.909 (0.878–0.930)***	0.716 (0.649–0.770)***
Psychological health	0.772 (0.720–0.811)***	0.534 (0.385–0.685)***
Social relationship	0.664 (0.458–0.755)***	0.583 (0.300–0.672)***
Spirituality	0.759 (0.597–0.819)***	0.548 (0.320–0.532)***

All significant with ***p < 0.001

### Multivariable linear regression analysis

To assess the association between WHOQOL HIV Bref domains and CD-4 count; linear regression test was applied (Table 7). Results showed when all domains were valued together; only physical, psychological and environment health domains were significantly associated with higher CD4 T-cell count (Beta = 0.121 (0.072–0.170), *p* < 0.001, Beta = 0.103 (0.037–0.168), *p* = 0.002, and Beta = 0.032 (− 0.120 to − 0.006), *p* = 0.032).

**Table 7:** Linear Associations of the WHOQOL HIV Bref with CD-4T cells

Predictors	Beta (95% CI)	*p* value
Physical health	0.121 (0.072 to 0.170)	< 0.001
Psychological health	0.103 (0.037 to 0.168)	0.002
Level of independence	0.030 (− 0.023 to 0.83)	0.260
Social relationships	0.036 (− 0.018 to 0.090)	0.190
Environment health	− 0.063 (− 0.120 to − 0.006)	0.032
Spiritual health	0.002 (− 0.120 to 0.041)	0.908

Dependent variable CD-4T cell count, all significant with *p* < 0.05

### Factor validity

Twenty-nine items of the WHOQOL HIV Bref instrument were analyzed for factor analysis. Kaiser-Meyer Olkin and Bartlett’s test of sphericity results were significantly acceptable. The analysis was acceptable as factor analysis of six domains with Eigen value above one represented 62.1% of the variance. First domain attributed to 34.5% of the variance. Furthermore, all six domains contain three or more items except for the fifth and sixth domains which include 2 and 1 items respectively. As recommended by Bryant et al. every domain should contain a minimum of three items; therefore, the model was modified to five domains [41]. Factor analysis of 5 domains accounted for 61% of the total variance. All unique areas were divided into a few aspects; however, similar regions were gathered, and stacked onto comparable components. For example, all items of the level of independence and physical health loaded into factor 1. All factors with details are given in Table 8.

**Table 8:** Exploratory factor analysis by varimax rotation with Kaiser’s normalization

Items	Original domain	Related factor loading				
		F^a^. 1	F. 2	F. 3	F. 4	F. 5
*Physical health*						
Bothered by HIV symptoms	Phy^b^	.926				
Pain and discomfort	Phy	.885				
Sleep and rest	Phy	.827				
Energy and fatigue	Phy	.617			.309	
Dependence on medical treatment	Independent^c^	.832				
Activities of daily living	Independent	.618				
Work capacity	Independent	.593				
Mobility	Independent	.804				
Bodily image and appearance	Psycho^d^	.816				
Physical safety and security	Environm^e^	.635				
*Environmental domain*						
Home environment	Environm		.603			
Accessibility of health services	Environm		.763			
Transport	Environm		.646		.483	
Physical environment	Environm		.569			
*Spirituality domain*						
Concerns about the future	Spiritual^f^			.856		
Worry about death	Spiritual			.809		
Forgiveness and blame	Spiritual			.710		
Concentration ability	Psycho			.318		
Meaningful life	Spiritual			.343		
*Social domain*						
Social support from friends	Social^g^				.665	
Positive feelings	Psycho	.555			.325	
Financial resources	Environm				.681	
Information for daily living	Environm				.665	
Sexual satisfaction	Social	.420	.430		− .355	
Social acceptance (inclusion)	Social				.452	
*Psychological health*						
Personal relationships	Social		.349			.804
Self-satisfaction	Psycho	.445				.554
Negative feelings	Psycho					.768
Participation in leisure activities	Environm					.606

Kaiser Kaiser–Meyer Olkin result 0.87 and Bartlett's test of sphericity result *p* < 0.0001

^a^F = factor loading

^b^Phy = physical domain

^c^Independent = level of independence domain

^d^Psycho = psychological domain

^e^Environm = environment domain

^f^Spiritual = spiritual domain

^g^Social = social domain

## Discussion

The current study focused on the appraisal of reliability, validity and cross-cultural adaptation of the Urdu version of the WHOQOL HIV Bref instrument. Findings demonstrate that WHOQOL HIV Bref is a highly reliable and validated tool in the Urdu language. This is the first study that systematically translated and validated the 31-item instrument in Pakistan. Conceptually our study highlighted the high reliability of the original instrument developed by WHO. The none response rate in our study was less (2.6%) displayed good acceptability by PLWHA. Overall, Cronbach’s alpha value is greater than 0.93 which indicates excellent internal consistency [42]. The percentage of the lowest and highest possible floor and ceiling scores of all domains in each domain was within an acceptable range, i.e. less than 10% [43]. Individual domains and each question’s internal consistency was more than 0.9, which is highly acceptable, and these findings are in line with the Iranian, Brazilian and Chinese studies [12, 18, 25]. The skewness and kurtosis value of almost all the items fall within the acceptable range (− 1.00 to 1.00) which are consistent with the findings of the study conducted among Taiwanese and older Portuguese PLWHA [20, 21].

Reliability coefficients (ICC values) of the Urdu version of the WHOQOL HIV Bref instrument ranging from 0.872 to 0.992; these findings are consistent with psychometric evaluation studies conducted in China and Malaysia [12, 16, 23]. The time interval was 12 to 16 days between the first and second ratings as proposed by Anne Anastasi and Susana Urbina [44]. The two weeks gap was selected to minimize the memory effect if the period is too short and that genuine differences in scores are not likely to have occurred if the period is too long [44].

Known group comparison shows the Urdu version of WHOQOL HIV Bref is a flexible tool for measuring the HIV care-related quality of life, as the questionnaire significantly differentiates btween patients with different levels of CD4 T cells; the higher the CD4 T cell levels, the better quality of life. These results are comparable to the findings of a study conducted in Chinese PLWH [12]. Of note, the difference in social relationship and spirituality facets (larger standard deviation) among PLWH with different HIV stages, indicates that though AIDS is a non-mortal disease at present, the level of affected emotional distress remains the same even at entirely different stage of HIV. This eplicates the reality that, despite HIV/AIDS is presently a treatable syndrome, however, does not represent a loss of life sentence. The participants have difficulty coping with the disease and managing related emotional distress regardless of their medical stage.

In addition, the social relationship factor may face similar challenges, and therefore interventional studies aimed at improving emotional and social well-being are needed to clarify this aspect. Along with other difficulties limited financial resources; the social aspect mainly affects PLWHA care and this is of particular importance in developing countries. Our study also showed that asymptomatic patients had better health-related outcomes compared to symptomatic patients. Previous studies by Tesfye et al., Zhu et al., and Saddki et al. have found that asymptomatic PLWHA have better HRQol than symptomatic subjects do and WHOQOL-HIV Bref has an excellent ability to discriminate against the HIV stages [12, 23, 24].

Factor analysis (FA) of WHOQOL-HIV Bref showed a five-factor model structure which is in line with the Portuguese and Malaysian HIV patients [21, 23]. This is found to be maximum about imbricate constructs between the factors due to the difference in perceptions and interpretations grounded in the Pakistani muslim culture. In addition to the Exploratory Factor Analysis (EFA), Canavarro et al. performed a WHOQOL-HIV Bref (CFA) jointly and found that each of the five-factor model and the original six-domain model worked reasonably well. [21]. Furthermore, multiple regression analyses from Peltzer et al. explained that only four domains (spirituality, psychological, surroundings, and level of independence) majorly predicted overall HRQoL [45]. Different studies also suggested that the model could be improved by changing some questions in a construct [21, 24].

WHOQOL Group Instruments have been identified as the best tools in terms of simplicity and scope of response [46]. Our findings suggest that WHOQOL HIV enables doctors and patients both to discuss more sensitive questions and to focus discussions on patient-induced non-medical issues. Our study has shown that WHOQOL-BREF is a sensitive tool for detecting PLWHA health variations in disease stages, viral load suppression and CD 4T lymphocytes count.

### Limitations

This research has limitations that need to be noted. First, Pakistan is a country of great diversity and ethnicity. There are more than 180,000 patients of HIV but only 22,947 people are receiving the ARV free of cost from the government established ART centers. Thus, the results of this study do not apply to those who are not receiving therapy. Seconed, most of the investigation populace included were intravenous drug users who were in a lamentable environment or incapable of sticking to the treatment rules.

Third, all study patients were outpatients and who might already be in a good physical state and none of the study participants had AIDS. Therefore, a discriminative property of instrument was tested between the symptomatic and asymptomatic groups only. For this reason, it is recommended that longitudinal studies should be conducted to further differentiate between different clinical stages of HIV infection. Finally, we focused only on the traditional psychometric properties of the Urdu version of the WHOQOL. Future studies are needed to examine the measurement error and clinimetric features (e.g., clinical validity and utility) of this rating scale.

## Conclusion

Current findings indicate that the Urdu version of WHOQOL HIV Bref is a psychometrically valid and culturally adapted HRQoL assessment tool for PLWHA in Pakistan. ART centres in Pakistan could use this tool to evaluate the HRQoL of HIV patients and develop tailored interventions to improve health outcomes.

## Abbreviations

HRQoL: Health-Related Quality of Life
HIV: Human Immunodeficiency Virus
AIDS: Acquired Immunodeficiency Syndrome
PLWHA: People Living With HIV/AIDS
HAART: Highly Active Anti-Retroviral Therapy
WHO: World Health Organization
NACP: National AIDS Control Program of Pakistan
REC: Research Ethics Committee
PIMS: Pakistan Institute of Medical Sciences
ICT: Islamabad Capital Territory
EFA: Exploratory Factor Analysis
ICC: Intraclass Correlation Coefficient
